# On the role of ocular torsion in binocular visual matching

**DOI:** 10.1038/s41598-018-28513-8

**Published:** 2018-07-13

**Authors:** Bernhard J. M. Hess

**Affiliations:** Department of Neurology, University Hospital Zurich, Zurich, CH-8091 Switzerland

**Keywords:** Motor control, Neurological disorders, Biomedical engineering

## Abstract

When an observer scans the visual surround, the images cast on the two retinae are slightly different due to the different viewpoints of the two eyes. Objects in the horizontal plane of regard can be seen single by aligning the lines of sight without changing the torsional stance of the eyes. Due to the peculiar ocular kinematics this is not possible for objects above or below the horizontal plane of regard. We provide evidence that binocular fusion can be achieved independently of viewing direction by adjusting the mutual torsional orientation of the eyes in the frontal plane. We characterize the fusion positions of the eyes across the oculomotor range by deriving simple trigonometric equations for the required torsion as a function of gaze direction and compute the iso-torsion contours yielding binocular fusion. Finally, we provide experimental evidence that eye positions in far-to-near re-fixation saccades indeed converge towards the predicted positions by adjusting the torsion of the eyes. This is the first report that describes the three-dimensional orientation of the eyes at binocular fusion positions based on the three-dimensional ocular kinematics. It closes a gap between the sensory and the motor side of binocular vision and stereoscopy.

## Introduction

**Figure 1:**
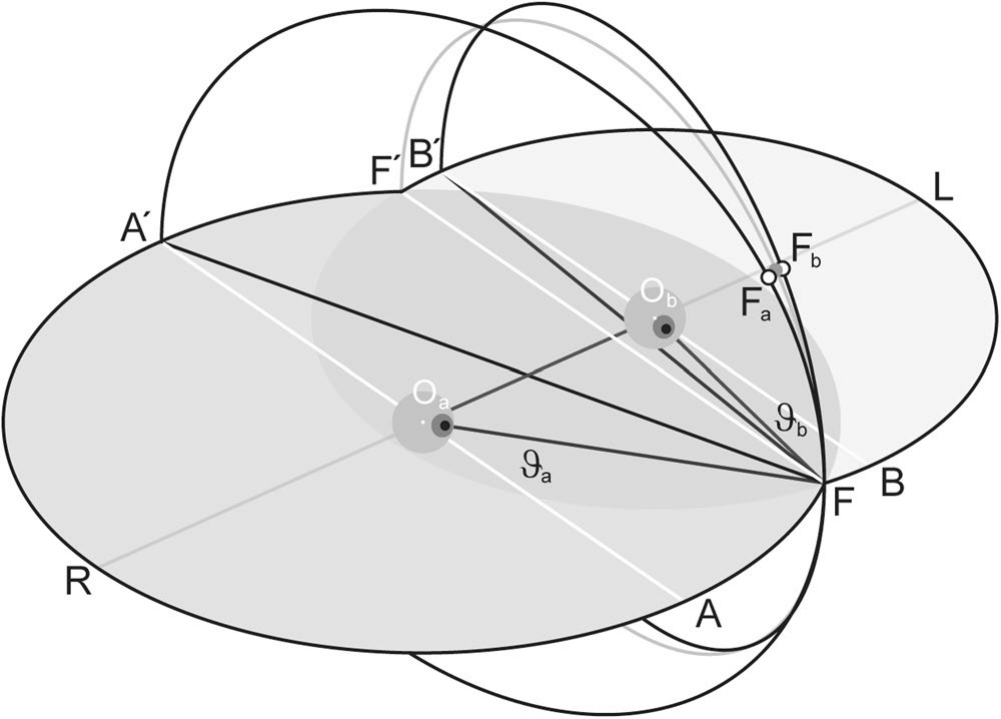
3D binocular kinematics during convergence. To fuse visual locations off the horizontal plane of regard, the eyes combine convergence (dis-conjugate rotations in the horizontal plane of regard) with compounded rotations in the respective vertical direction-planes (vertical planes through the lines A′F and B′F) and the common frontal plane (plane through O_a_ and O_b_). In convergent positions, the vertical rotations of the eyes move the fixation points away from the locus of positions that can be seen single (see gray circular segment through F′ and F versus black circular segments through A′, F_a_, F and B′, F_b_, F). We show algebraically that fusion of the diplopic fixation positions F_a_ and F_b_, can be achieved by small concerted rotations of the right and left eye in the frontal plane (fusion position represented as gray dot on the locus of points that can be seen single). Notice that, assuming a fixed distance, |O_a_O_b_| = 1, between the rotation centers, the distances a = |O_a_F| and b = |O_b_F| from those centers to the fixation point F are uniquely determined by eyes’ azimuths ϑ_a_ and ϑ_b_ (sine law). A′, B′, rear fixation points (occipital points of each eye’s spherical field of fixation, Helmholtz^23^); O_a_ A, O_b_ B reference directions of the right, left eye; gray-shaded circular areas, horizontal plane of regard; ϑ_a_, ϑ_b_, horizonal rotation angles. Sketch true to scale, drawn in units of interocular distance |O_a_O_b_|.

The retinal images of objects in visual space establish geometric correlations between slightly different views of the spherical visual field. To align the eyes on a visual object of interest, the brain first must establish a one-to-one correspondence between features of the projected retinal images and estimate relative distances. Cues about self-orientation, binocular parallax, imaging properties of the eyes and other factors make a clean geometric solution to the correspondence problem seemingly impractical. In contrast to the problem of depth estimation that has been shown to require extraretinal cues^1^, here we are solely concerned with a solution of the correspondence problem on the bases of low-level visual cues. Such cues have been shown to generate vergence eye movements without depth perception^2^. A first step towards such a solution was the geometric insight that those points in visual space that lie on a circle passing through the fixation point and joining the eyes’ nodal points cast their images on corresponding retinal points^3,4^. Although psychophysical studies showed that this notion was essentially true, at least in the horizontal plane of regard, attempts to extend the concept into the vertical dimensions of the binocular visual field remain unconvincing and controversial^5^. More recently, Schreiber and colleagues^6^ have presented a model of corresponding retinal positions by including the non-linear kinematics of three-dimensional eye movements. These authors presumed that images of binocular objects outside the horizontal plane of regard remain diplopic except for the median plane where the visual lines indeed may intersect. Recent experimental evidence obtained from far-to-near re-fixation saccades has shown that rhesus monkey can perfectly fuse targets outside the horizontal planes, a finding that challenged the widely held notion of corresponding retinal positions^7,8^. A fundamental difference between visually-guided eye movements in near and far vision is commutativity known as Donders law^9^: In the near visual space it matters whether the eyes move first horizontal and then vertical or vice versa. Since the resulting positions of each eye slightly differ in torsion the eyes must torque relative to each other to fuse diplopic targets. This torsion rotates the retinal images in the observer’s frontal plane while keeping the relative orientation of monocular image features invariant (Fig. 1).

**Figure 2:**
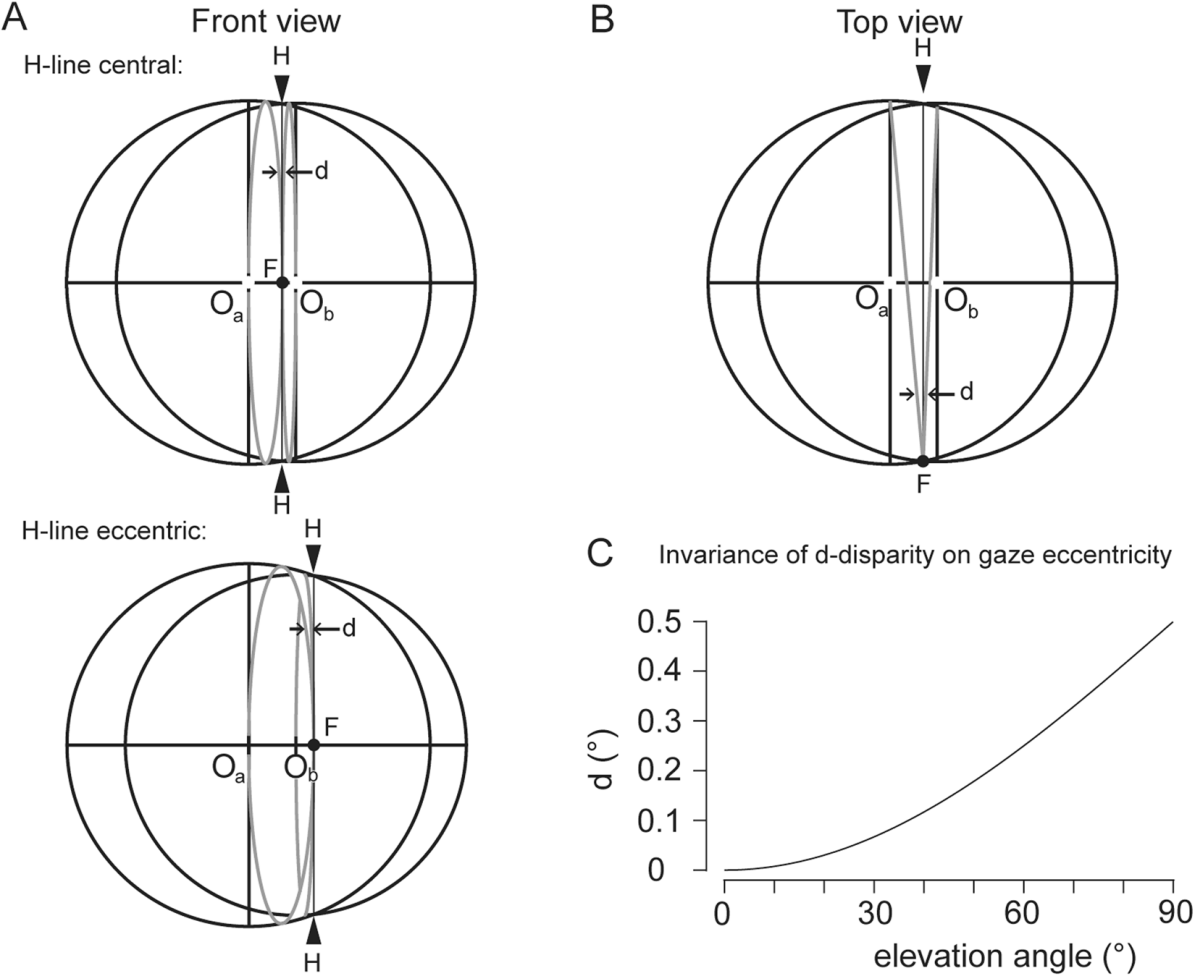
Front and top view onto spherical shells of constant target distances relative to the right (O_a_) and left (O_b_) ocular rotation centers. The shells intersect at a circle representing the geometric locus of points in space at equal distances from the two rotation centers (Helmholtz circles, projecting as straight lines through F, labelled by arrowheads H). Donders-Listing positions are positions on circles through F and the rear fixation points that project as vertical ellipses (in gray) in the two sketches of panel A and as straight lines in B (in gray). On one hand these circles are tangent to the median plane of each eye (projected as vertical lines through the rotation centers O_a_ and O_b_) and on the other to the Helmholtz circle (labelled by arrowheads H). Note that the shells intersect each other either between the eyes (H-line central) or off to one side (H-line eccentric). Convergence 15° in both cases. Binocular disparity was defined as the disparity between the right and left eye’s Donders-Listing positions (arrowheads labelled ‘d’ in panels A, B). In the horizontal plane of regard (projected as horizontal line joining O_a_ and O_b_), binocular disparity is zero in every gaze direction. Off this plane, it increases up to a maximum of 0.5, independent of the horizontal viewing direction and fixation depth; see horizontal disparity d plotted versus elevation in panel C.

Although the configuration space of ocular rotations is in general three-dimensional (six-dimensional considering binocular eye movements), the problem of retinal correspondence can be illustrated in a simple two-dimensional sketch depicting a head-fixed binocular ‘retinal’ map in the observer’s frontal plane (Fig. 2A). In this map the motion of the images of a single point in visual space, imagined as small spot at the center of the entrance pupils of the observer (with the head still and upright), would follow typically straight lines during most eye movements except for eye movements between or towards objects in near visual space. For example, when making a saccade from an object at near distance (black dot F, Fig. 2A) to a target straight up in the visual field, the eyes initially would tend to follow curved paths as indicated by the two ellipses in gray in Fig. 2A. These curved paths reflect torsion-free motions of the eyes from a common secondary position to tertiary positions, also called Donders-Listing positions. To finally reach a target straight up on the depicted vertical line, tangent to the two ellipses, the eyes must rotate in the frontal plane to compensate for the disparate elliptic paths. This tangent line that represents the projection of binocular positions in space that can be seen single has been called the Helmholtz line or H-line for short. The landing positions of far-to-near re-fixation saccades have been reported to closely scatter around the corresponding H-line^7^ suggesting in fact that binocular disparity can be defined as the difference between the Donders-Listing positions of the eyes (see arrows labelled ‘d’, Fig. 2). Interestingly such a definition would be independent of the depth plane of fixation (Fig. 2C). The reason is that the minor axes of the two ellipses invariantly span the baseline between the two ocular rotation centers whereas the two semi-minor axes span half the baseline, independent of where the near fixation point lies in the horizontal plane (compare upper and lower panel in Fig. 2A).

Using this novel concept of disparity, we show that indeed the eyes can perfectly fuse retinal images of objects across the whole binocular visual field with virtually zero binocular disparity. The underlying geometry relies on the experimentally well-established kinematics of Donders-Listing eye movements. We show that binocular disparity is based on the interplay of the iso-disparity maps that are identical for each eye and thus by themselves independent of the depth plane of fixation. The one single parameter (apart from ocular torsion) that controls binocular disparity is the angle of convergence of the eyes, which depends on the estimated depth plane of fixation. Over a large portion of the visual field binocular disparities can be eliminated by a moderate adjustment of the torsional stance of the eyes. We verified these novel concepts by comparing target fusion in far-to near re-fixation saccades in non-human primates with the predictions based on torsional fusion of diplopic images.

## Methods

### General eye movements

We determined a head-fixed three-dimensional Cartesian coordinate system using the direction straight ahead of each eye and the line joining the right and left eye’s rotation center to define the unit vectors ${\hat{e}}_{1}$, ${\hat{e}}_{2}$ and ${\hat{e}}_{3}={\hat{e}}_{1}\times {\hat{e}}_{2}$(x, cross product). We shell denote unit vectors in normal font with a caret and all other vectors in bold font. Rotations in the horizontal, vertical and frontal plane (orthogonal to straight ahead) are abbreviated by R_H_, R_V_, and R_F_.

A general ocular rotation or eye position was defined as a torsion-free horizontal-vertical rotation, also called a Donders-Listing rotation (denoted by R_DL_), followed by a rotation in the frontal plane. Specifically, we considered the general rotations *R*_*F*_*R*_*DL*_ = *R*_*F*_*R*_*v*_*R*_*H*_ and *R*_*F*_*R*_*DL*_ = *R*_*F*_*R*_*h*_*R*_*V*_, where R_v_ and R_h_ (with lower case subscripts) denoted the vertical and horizontal rotations in the respective direction-planes. The resulting gaze directions were calculated by conjugation of the reference gaze direction ${\hat{g}}_{0}={\hat{e}}_{1}$ with the rotation sequence, for example $\hat{g}={R}_{F}{R}_{DL}{\hat{g}}_{0}{R}_{DL}^{-1}{R}_{F}^{-1}={R}_{F}\hat{d}{R}_{F}^{-1}$. Unit gaze directions are denoted by $\hat{g}={\sum }_{i}{g}_{i}{\hat{e}}_{i}$ and unit Donders-Listing directions by $\hat{d}={\sum }_{i}{d}_{i}{\hat{e}}_{i}$. We used the subscripts ‘a’ and ‘b’ to distinguish variables and coefficients related to the right and left eye, respectively. The explicit evaluation of a general eye position of the right and left eye yielded the gaze vectors ${\hat{g}}_{a}$ and ${\hat{g}}_{b}$ (see Supplementary, General eye position):

1a $${\hat{g}}_{a}={R}_{F}{\hat{d}}_{a}{R}_{F}^{-1}={Q}_{a}{\hat{e}}_{1}+\{{S}_{a}\,\cos \,{\xi }_{a}+{R}_{a}\,\sin \,{\xi }_{a}\}{\hat{e}}_{2}-\{{R}_{a}\,\cos \,{\xi }_{a}-{S}_{a}\,\sin \,{\xi }_{a}\}{\hat{e}}_{3}$$

1b $${\hat{g}}_{b}={R}_{F}{\hat{d}}_{b}{R}_{F}^{-1}={Q}_{b}{\hat{e}}_{1}+\{{S}_{b}\,\cos \,{\xi }_{b}+{R}_{b}\,\sin \,{\xi }_{b}\}{\hat{e}}_{2}-\{{R}_{b}\,\cos \,{\xi }_{a}-{S}_{b}\,\sin \,{\xi }_{b}\}{\hat{e}}_{3}$$

here ${\hat{d}}_{a}={R}_{DL}{\hat{g}}_{0}{R}_{DL}^{-1}={({Q}_{a},{S}_{a},-{R}_{a})}^{T}$ and ${\hat{d}}_{b}={R}_{DL}{\hat{g}}_{0}{R}_{DL}^{-1}={({Q}_{b},{S}_{b},-{R}_{b})}^{T}$ were the Donders-Listing directions of the right and left eye (the superscript ‘T’ stands for transpose) with components *Q*_*a*_ = cos*ϑ*_*a*_cos^2^*η*_*a*_/2 − sin^2^*η*_*a*_/2, *R*_*a*_ = sin*η*_*a*_cos*ϑ*_*a*_/2, *S*_*a*_ = sin*ϑ*_*a*_cos^2^*η*_*a*_/2 and *Q*_*b*_ = cos*ϑ*_*b*_cos^2^*η*_*b*_/2 − sin^2^*η*_*b*_/2, *R*_*b*_ = sin*η*_*b*_cos*ϑ*_*b*_/2, *S*_*b*_ = sin*ϑ*_*b*_cos^2^*η*_*b*_/2, respectively. The angles ϑ_a_, η_a_ and ϑ_b_, η_b_ denoted the rotation angles in the horizontal and vertical direction-planes of the right and left eye, respectively. The angles ξ_a_ and ξ_b_ denoted the respective rotation angles in the frontal plane.

To study the binocular aspects of fixations, we used a head-fixed coordinate system X-Y-Z with origin centered at the right eye’s rotation center, the x-axis aligned with the direction straight ahead and the y-axis joining the right and left eye’s rotation centers. We assumed that the rotation center of the left eye was at unity distance from the right eye and that the optical centers of the eyes coincided with the rotation centers. In this system, the geometric conditions for the gaze vectors ***g***_*a*_ = (*g*_*aX*_, *g*_*aY*_, *g*_*aZ*_)^*T*^ and ***g***_*b*_ = (*g*_*bX*_, *g*_*bY*_, *g*_*bZ*_)^*T*^ to meet were *g*_*aX*_ = *g*_*bX*_, *g*_*aY*_ = *g*_*bY*_ + 1 and *g*_*aZ*_ = *g*_*bZ*_, using the assumption that the lateral separation of the eyes corresponded to the unity of the X-Y-Z coordinate system. We thus obtained the following set of linear equations.

2a $${({g}_{aY},{g}_{aZ})}^{T}=a{R}_{F}({\xi }_{a}){({R}_{a},{S}_{a})}^{T}$$

2b $${({g}_{bY},{g}_{bZ})}^{T}=b{R}_{F}({\xi }_{b}){({R}_{b},{S}_{b})}^{T}+{(1,0)}^{T}$$

here *R*_*F*_(*ξ*_*a*_) and *R*_*F*_(*ξ*_*b*_) described the rotations in the frontal plane (Y-Z plane) through the angles ξ_a_ and ξ_b_ as described in equation 1a and b. These rotations left the x-component of the gaze vectors invariant. The solution can be expressed in the following two chiral symmetric forms (see Supplementary).

3a $$(\begin{array}{c}\sin \,{\xi }_{a}\\ \cos \,{\xi }_{a}\end{array})=\{{p}_{a}{\rm{\Lambda }}(\begin{array}{c}\sin \,{\xi }_{b}\\ \cos \,{\xi }_{b}\end{array})+{q}_{a}(\begin{array}{c}{R}_{a}\\ {S}_{a}\end{array})\}$$

3b $$(\begin{array}{c}\sin \,{\xi }_{b}\\ \cos \,{\xi }_{b}\end{array})=\{{p}_{b}{{\rm{\Lambda }}}^{-1}(\begin{array}{c}\sin \,{\xi }_{a}\\ \cos \,{\xi }_{a}\end{array})-{q}_{b}(\begin{array}{c}{R}_{b}\\ {S}_{b}\end{array})\}$$

where ${p}_{a}=(b/a)\sqrt{D}/({R}_{a}^{2}+{S}_{a}^{2})$, ${p}_{b}=(a/b)\sqrt{D}/({R}_{b}^{2}+{S}_{b}^{2})$, ${q}_{a}=(1/a)/({R}_{a}^{2}+{S}_{a}^{2})$,${q}_{b}=(1/b)/({R}_{b}^{2}+{S}_{b}^{2})$. The matrix $${\rm{\Lambda }}=(\begin{array}{cc}\cos \,{\sigma }_{ba} & \sin \,{\sigma }_{ba}\\ -\,\sin \,{\sigma }_{ba} & \cos \,{\sigma }_{ba}\end{array})$$ was a rotation matrix with $\cos \,{\sigma }_{ba}=({R}_{b}{R}_{a}+{S}_{b}{S}_{a})/\sqrt{D}$, $\sin \,{\sigma }_{ba}=({S}_{b}{R}_{a}-{R}_{b}{S}_{a})/$ $\sqrt{D}$ and $D=({R}_{a}^{2}+{S}_{a}^{2})({R}_{b}^{2}+{S}_{b}^{2})$. The angles σ_ab_ = σ_ba_ were symmetric functions of the right and left eyes’ rotation angles. Each of these equations described one eye’s torsion as a function of the other eye’s torsion that was required for the gaze lines to meet at near distance. The six coefficients Q_a_, R_a_, S_a_ and Q_b_, R_b_, S_b_ depended on the horizontal and vertical rotation angles ϑ_a_, η_a_, and ϑ_b_, η_b_ of the respective eye (see equation 1a,b). To solve for the torsion angles, we wrote equation 3a as

4a $$\sin ({\xi }_{b}+{\sigma }_{ba}+{\sigma }_{a})=\{1-{{p}_{a}}^{2}-{{q}_{a}}^{2}({{R}_{a}}^{2}+{{S}_{a}}^{2})\}/2{p}_{a}{q}_{a}\sqrt{{{R}_{a}}^{2}+{{S}_{a}}^{2}}$$

with $\sin \,{\sigma }_{a}={S}_{a}/\sqrt{{R}_{a}^{2}+{S}_{a}^{2}}$, $\cos \,{\sigma }_{a}={R}_{a}/\sqrt{{R}_{a}^{2}+{S}_{a}^{2}}$ and likewise equation 3b as

4b $$\sin ({\xi }_{a}-{\sigma }_{ab}+{\sigma }_{b})=\{1-{{p}_{b}}^{2}-{{q}_{b}}^{2}({{R}_{b}}^{2}+{{S}_{b}}^{2})\}/(2{q}_{b}{p}_{b}\sqrt{{{R}_{b}}^{2}+{{S}_{b}}^{2}})$$

with $$\sin \,{\sigma }_{b}={S}_{b}/\sqrt{{R}_{b}^{2}+{S}_{b}^{2}}$$, $$\cos \,{\sigma }_{b}={R}_{b}/\sqrt{{R}_{b}^{2}+{S}_{b}^{2}}$$.

The ‘binocular’ angles σ_ab_ and σ_ba_ = σ_ab_ were related to the ‘monocular’ angles σ_a_ and σ_b_ by cos*σ*_*ab*_ = cos(*σ*_*a*_ − *σ*_*b*_) and sin*σ*_*ab*_ = sin(*σ*_*b*_ − *σ*_*a*_).

The model’s solutions encompassed two classes of fusional torsions of the eyes: One class of torsions consisted of binocular torsions of opposite signs fusing near targets in the visual field between the meridian planes of the eyes (see Fig. 2A, upper panel). The other class consisted of torsions of equal signs fusing near targets off on either side of the central binocular field (see Fig. 2A, lower panel). A useful criterion for handling numerical ambiguities that could arise at the transition zones was to check the mutual consistency of the binocular relations cos(σ_ab_) and sin(σ_ab_).

### Projected retinal images and disparity vectors

To determine whether the eyes indeed left the Donders-Listing positions during fusion of near targets, we measured the differences between the Donders-Listing and the predicted fusion positions (see Figs 1 and 2). The disparities of fixation positions were *δy* = *d*_*aY*_ − *d*_*bY*_ − 1 = (*d*_*aY*_ − *g*_*aY*_)−(*d*_*bY*_ − *g*_*bY*_) and *δz* = *d*_*aZ*_ − *d*_*bZ*_ = (*d*_*aZ*_ − *g*_*aZ*_) − (*d*_*bZ*_ − *g*_*bZ*_) in the horizontal and vertical direction, respectively. In projective coordinates, the Donders-Listing and the fusion positions were ${\bar{{\boldsymbol{d}}}}_{a}={{\boldsymbol{d}}}_{a}/{d}_{aX}$, ${\bar{{\boldsymbol{g}}}}_{a}={{\boldsymbol{g}}}_{a}/{g}_{aX}$ for the right eye and ${\bar{{\boldsymbol{d}}}}_{b}={{\boldsymbol{d}}}_{b}/{d}_{bX}$, ${\bar{{\boldsymbol{g}}}}_{b}={{\boldsymbol{g}}}_{b}/{g}_{bX}$ for the left eye. Since mutual equality of the forward components was a necessary condition for fusion (see equation 2), we used the following natural definition of binocular disparity.

5a $$\delta \bar{y}=({\bar{d}}_{ay}-{\bar{g}}_{ay})-({\bar{d}}_{by}-{\bar{g}}_{by})$$

5b $$\delta \bar{z}=({\bar{d}}_{az}-{\bar{g}}_{az})-({\bar{d}}_{bz}-{\bar{g}}_{bz})={\bar{d}}_{az}-{\bar{d}}_{bz}$$

These definitions make metrically sense because they are independent of the depth plane of fixation. The symmetry of these definitions with respect to the fusion positions suggested that it suffices to analyze the disparities with respect to one eye, say the right eye, which boil down to the relations $\delta {\bar{y}}_{a}={\bar{d}}_{ay}-{\bar{g}}_{ay}$ and $\delta {\bar{z}}_{a}={\bar{d}}_{az}-{\bar{g}}_{az}$. The polarity of $\delta {\bar{y}}_{a}$ was negative in the left and positive in the right visual hemi-field. In contrast the polarity of $\delta {\bar{z}}_{a}$ was positive in the upper and negative in the lower hemi-field.

For numerical simulations, we chose an oculomotor range of ±40° with a resolution of 0.5° in horizontal and vertical direction (161 × 161 points). We computed the disparities of the right eye across its whole oculomotor range. Numerically, fusion was accepted at the level |*g*_*aY*_ − *g*_*bY*_ − 1| < 2⋅10^−15^ and |*g*_*aZ*_ − *g*_*bZ*_| < 2⋅10^−15^. These levels referred to the fixations in visual space before projection to the image space. They depended on the chosen resolution, which could be pushed down to machine resolution. Since the left eye’s disparity pattern was mirror-symmetric to that of the right eye with respect to the z-axis we considered only the pattern of the right eye. We expressed the disparities as functions of the projected fusion positions $({\bar{g}}_{ay},{\bar{g}}_{az})$ and solved the equations $\delta {\bar{y}}_{a}=c$ and $\delta {\bar{z}}_{a}=c$ numerically for a set of values of the constant c. In this way we obtained contour lines of equal disparity, which we plotted against the projected fusion positions. The distance between contour lines was 10^−4^ and the lines’ half-width was 2∙10^−5^. Analogously, we expressed the torsion of the right eye as a function of the projected fusion positions and solved the equations *ξ*_*a*_ = *c* for a set of values of the constant c to obtain contour lines of equal torsion. These lines described the amount of torsion required for fusion at the respective fusion positions $({\bar{g}}_{ay},{\bar{g}}_{az})$, which were the same positions used to determine disparities. Torsional contour lines were computed every 0.1° using a half-width of 0.025°. Because of the translation-symmetry of the disparity patterns the calculations yielded identical results for the right and the left eye.

### Experimental fusion positions during far-to-near re-fixation saccades

To test the torsional fusion model described so far, we used three-dimensional eye movement data from behaviorally trained non-human primates that had been obtained in accordance with the recommendations in the Guide for the Care and Use of Laboratory Animals of the US National Institutes of Health. Results obtained from analyses of different aspects of these eye movement data have been reported in earlier studies. The housing, husbandry and experimental procedures had been reviewed, approved and supervised by the Veterinary Office of the Canton of Zurich as also reported earlier^7^. Here we used these same data to compare the fusion positions predicted by the model with the actual trajectories and the final landing positions of far-to-near re-fixation saccades. In brief, we re-analyzed the eye movement data from three rhesus monkeys that had been trained to make re-fixation saccades from a small LED target 80 cm straight ahead to a near target about 10° above or below the horizontal plane of regard. We had previously shown that the end positions of these saccades closely scattered around positions on the Helmholtz circle, which allows binocular single vision. As described here and in the previous publications we expressed the 3D saccadic movements as Donders-Listing rotations followed by torsional rotations in the frontal plane that is by the relation ${R}_{exp}={R}_{F}{R}_{DL}$ as described. We calculated the respective experimental gaze positions ${\hat{g}}_{\exp }={R}_{\exp }{\hat{g}}_{0}{R}_{exp}^{-1}$ and the Donders-Listing positions ${\hat{d}}_{\exp }={R}_{{\rm{DL}}}{\hat{g}}_{0}{R}_{DL}^{-1}$. Next, we factorized the Donders-Listing rotations into a horizontal rotation followed by a vertical rotation and determined the rotation angles and the dihedral angles subtended by the median vertical plane and the respective vertical direction-planes. With these angular parameters at hand, we solved equation 4a and b for the rotation angles of ocular torsion. Finally, we solved equation 1a and b for the predicted fusion positions of the right and left eye. We denoted these fusion positions by ${\hat{f}}_{a}$ and ${\hat{f}}_{b}$ in order to distinguish them from the far-to near saccade trajectories ${\hat{g}}_{a}\equiv {\hat{g}}_{a.\exp }$ and ${\hat{g}}_{b}\equiv {\hat{g}}_{b.\exp }$. To keep notations simple, we will drop the subscript add-on ‘exp’ if the experimental context is clear. Finally, we determined the horizontal and vertical disparities $\delta {\bar{y}}_{a}={\bar{f}}_{ay}-{\bar{g}}_{ay}$ and $d{\bar{z}}_{a}={\bar{f}}_{az}-{\bar{g}}_{az}$, expressed as projections of the theoretical and experimental trajectories as previously defined. For further analysis, we accepted only trials with horizontal disparities $\delta {\bar{y}}_{a}\, < \,{10}^{-3}$, corresponding to a deviation of ~1.5 arc min from the geometrically correct angle in symmetric vergence of 15°.

## Results

**Figure 3:**
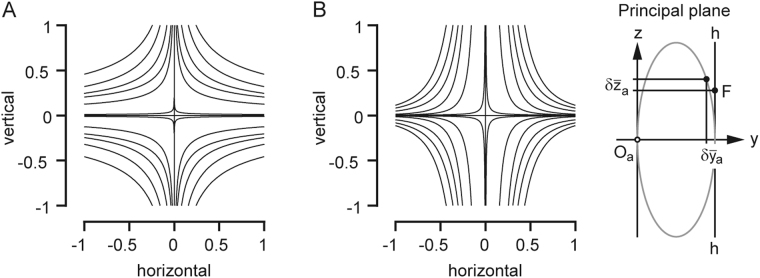
(**A**,**B**) Horizontal and vertical iso-disparity contours plotted against the projected fusion positions. The inset ‘Principal plane’ illustrates graphically the definition of horizontal ($\delta {\bar{y}}_{a}$) and vertical ($\delta {\bar{z}}_{a}$) disparity of the projected right retinal image. Disparities were zero in the direction straight ahead (primary direction) and in secondary horizontal and vertical directions. The weight of the first contour was 10^−4^; the subsequent contours were spaced evenly in steps of 5∙10^−4^ (relative to the center); contour-width was ±2∙10^−5^. Horizontal disparities (panel A) were negative in the left and positive in the right visual hemi-field. Vertical disparities (panel B) were positive in the upper and negative in the lower hemi-field. Disparity contours did not depend on the vergence angle. Average parameters (±1SD) of the fitted family of hyperbola-like functions *z* = *u*^2^*y*^−*v*^(y > 0) were u = 0.48 (±0.11), v = 0.54 (±0.03), r^2^ = 0.9998 (N = 25) for the horizontal disparity contours and u = 0.21 (±0.11), v = 2.7 (±0.29), r^2^ = 0.997 (N = 28) for the vertical disparity contours. Range tested ±40° × ±40°.

Our analysis of retinal correspondences showed that identical disparity patterns underlie the fusion of objects across the binocular visual space, reflecting the mirror symmetry of the visual field with respect to the median plane. We computed iso-disparity contours by evaluating the disparity vector of binocular single positions across the oculomotor field (Fig. 3). The contours represented curves of constant horizontal and vertical disparity between the projected Donders-Listing and the fusion positions. Based on the definitions, the contours of horizontal iso-disparity were positive in the right and negative in the left visual hemi-field. Similarly, the vertical iso-disparity contours were positive in the upper and negative in the lower hemi-field. In both cases the weights of the contours increased with radially increasing distance from the origin. Since the iso-disparity contours were plotted against the projected fusion positions ${\bar{g}}_{ay}={g}_{aY}/{g}_{aX}$ and ${\bar{g}}_{az}={g}_{aZ}/{g}_{aX}$ (in right eye-centered coordinates), their shapes and weights were invariant against the state of ocular convergence (see Fig. 2).

We have fitted the underlying data by rectangular hyperbola-like functions, i.e. curves of the form *z* = *u*^2^*y*^−*v*^(for y > 0) where ‘u’ was the vertex-parameter (analogous to the vertex of a rectangular hyperbola) and the exponent ‘v’ was a positive fractional exponent (instead of 1 as for a rectangular hyperbola). Due to the point-symmetry of the contour patterns with respect to the center of rotation we fitted the level curves in one quadrant by taking only contours with at least three data points into account. The fits of horizontal (N = 25) and vertical disparity contours (N = 28) had mean coefficients of variation of 0.9998 and 0.997. The average vertex parameters (±1SD) of the family of horizontal and vertical disparity-contours were 0.48 (±0.1) and 0.21 (±0.1) and the average exponents were 0.54 (±0.03) and 2.7 (±0.3). It can be showed that the horizontal disparity contours are much more sensitive to changes across the span of its parameters than the vertical disparity contours.

### Binocular disparity patterns

The following rationale provides a synthesis between the individually projected images of the eyes: On one hand it is safe to assume that the projected images of objects at optical infinity superimposed one-to-one at superordinate central levels. On the other hand, we know that the distances from the eyes’ rotation centers to objects in near visual space are related to the eyes’ azimuths during convergence (see triangle O_a_O_b_F in Fig. 1). Specifically, the distance from the right eye to the fixation object is ${g}_{aX}=a\,\cos \,\alpha$ with $a=\cos \,\beta /\sin (\alpha -\beta )$ for symmetric convergence of the eyes. Hereby the angles α and β are the complementary angles between the respective gaze lines and the line joining the rotation centers. In the projection plane, the constraint Δ = *g*_*aY*_ − *g*_*bY*_ = 1 used for deriving the fusion equations (Methods, equation 2) must therefore be replaced by the relation $\bar{{\rm{\Delta }}}={\rm{\Delta }}/{g}_{aX}$: The farther the fixation objects, the closer the optical centers of the images in the projection plane. We shell call the superordinate projection plane the principal plane for short. Thus, we obtained a lateral separation of the contour maps in this plane of $\bar{{\rm{\Delta }}}={\rm{\Delta }}/{g}_{aX}=\sin (\alpha -\beta )/(\cos \,\alpha \,\cos \,\beta )$. In case of symmetric vergence, iso-disparity lines of equal weights met at a vertical line at equal distances from the rotation centers. This line, H-line called for short, corresponded to the projection of the Helmholtz-circle of binocular single positions onto the principal plane (see gray circle through F’ and F, Fig. 1). In asymmetric convergence, the iso-disparity contours that meet at the H-line do have different weights. For example, the horizontal iso-disparity contours of the eye closer to the H-line do meet iso-disparity contours of higher weights of the eye farther away from the H-line. In case the H-line coincided with the median plane of one eye, that is, when the binocular single positions were in primary or secondary vertical positions of one eye the model predicted zero contribution of this eye to binocular disparity and maximal contribution of the other eye. In general, the predicted binocular disparities modulated much less in horizontal than in vertical gaze directions as the following analysis shows.

Evaluation of binocular disparities as functions of gaze directions (relative to the right eye) yielded the relations $\delta \bar{y}=(a{S}_{a}-b{S}_{b}-1)/a{Q}_{a}$ and $\delta \bar{z}=(\,-\,a{R}_{a}+b{R}_{b})/a{Q}_{a}$(see Methods, equations 1 and 5). From the condition of zero horizontal disparity $\delta \bar{y}=0$ followed that $a{S}_{a}-b{S}_{b}=1$. Similarly, from the condition of zero vertical disparity $\delta \bar{z}=0$ followed *bR*_*b*_ = *aR*_*a*_. Thus, the fusion model predicted that binocular disparity was zero during versions movements as expected. Secondly, it predicted that during convergence binocular disparity could only be zero for eye movements in the horizontal plane of regard. Outside this plane the eyes would have to torque to achieve binocular fusion of targets (for details see Supplementary Table S1).

To describe the disparity patterns more specifically, we evaluated the predicted binocular disparities $\delta \bar{y}$ and $\delta \bar{z}$ numerically across the binocular visual field. These calculations depended on the azimuths and on just one of the vertical rotation angles of the eyes. In fact, the model required that the vertical angles were related to each other in fusion by the eyes’ azimuths, a finding that embodies Hering’s law of equal innervation^10^ in kinematic terms (for details see Supplementary Information).

**Figure 4:**
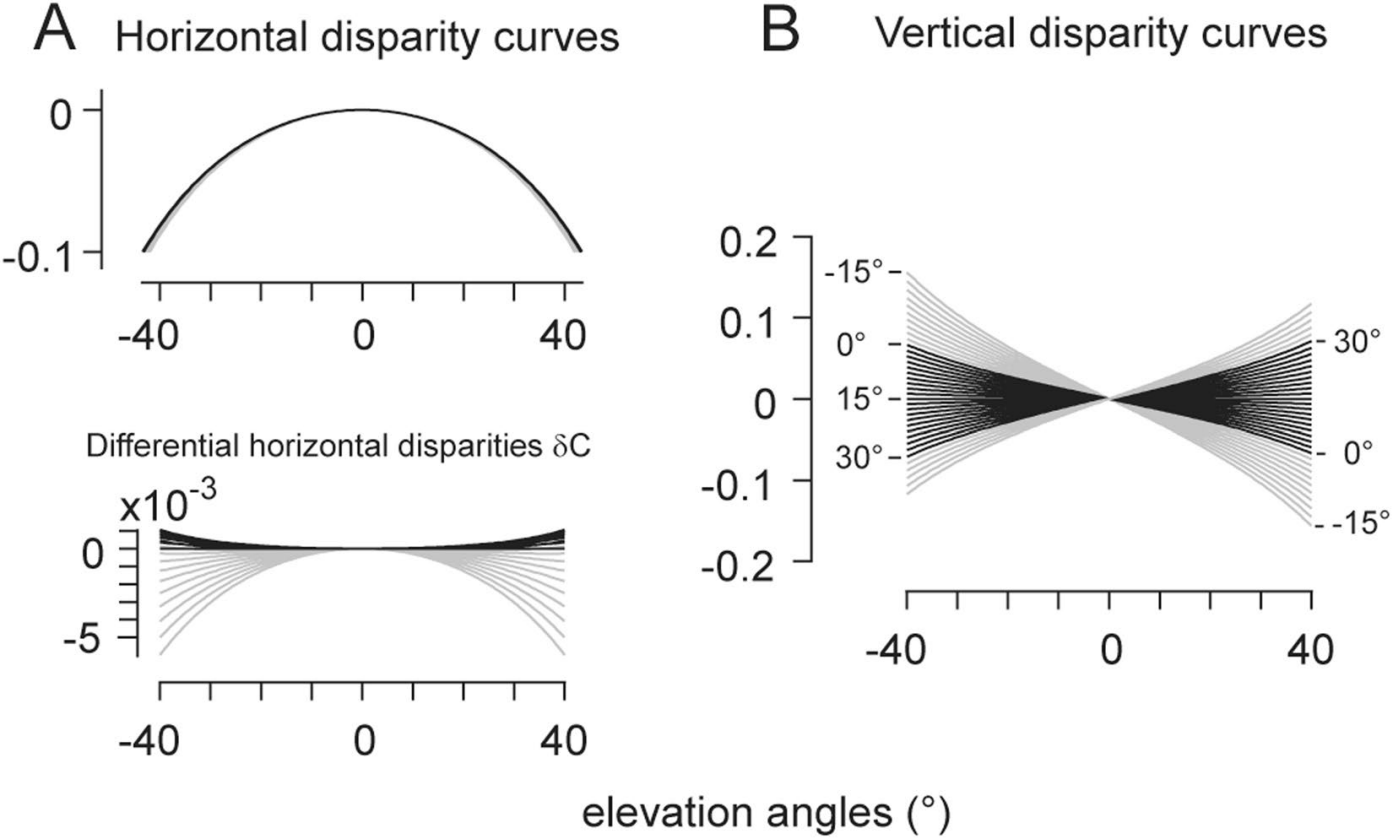
Horizontal and vertical binocular disparities relative to the right eye at constant convergence. (**A**) Family of horizontal disparity curves parameterized by azimuth angles varying between −15° to 15° in steps of 1.5° (21 curves superimposed) and plotted as functions of vertical elevations in the range of −40° to 40°. Convergence was 30° (azimuth of left eye thus ranged from −45° to −15°). Disparities were negative in all four quadrants of the visual field. *Panel A, lower half*: Differential horizontal disparities δC_i_ = C_i_-C_0_ (i = 1 to 21), computed relative to the horizontal disparities in symmetric convergence (C_0_). Differences were positive in the central binocular field (black curves) indicating decreasing curvatures towards the borders of the field. Outside this field curvatures increased (gray curves). Note that in this plot the disparity weights were weighted ×10^−3^. (**B**) Family of vertical disparity curves, parameterized by azimuth angles varying between −15° to 35° in steps of 1.5°. Disparities at zero-azimuth were negative in the upper hemi-field (positive elevation angles) and positive in the lower hemi-field. Vertical disparities increased in absolute terms symmetrically towards the borders of the visual field (black curves: central binocular field). Disparities changed polarity with respect to the median plane (plane α = −β).

The evaluation of binocular disparity depended on the depth plane of fixation. For an illustration, we have chosen a moderate constant convergence of 30° and evaluated the equations in right eye coordinates by varying the azimuth from −15° to 15° (and thus the azimuth of the left eye from −45° to 15°) and vertical gaze direction from −40° to 40° (Fig. 4). The horizontal binocular disparities varied symmetrically relative to the horizontal plane of regard. In the central binocular field, defined as the visual field within the meridian planes of each eye, the disparity curves changed very little in shape from field border to field border (black curves, Fig. 4A). Outside this field, the curves’ curvature increased systematically (gray curves, Fig. 4A). Overall the deviations from the binocular disparity profile in symmetric convergence were less than about 1∙10^−3^ in the central binocular field (black curves in Fig. 4A, lower panel) and varied in a range of up to about 5∙10^−3^ outside this field (gray curves in Fig. 4A, lower panel). The vertical binocular disparities showed a more uniform behavior across the binocular field. Binocular vertical disparities were negative in the upper hemi-field and positive in the lower hemi-field. In absolute terms binocular vertical disparities increased strongly toward and beyond the borders of the central binocular field (compare black versus gray curves, Fig. 4B).

### Fusional torsion

**Figure 5:**
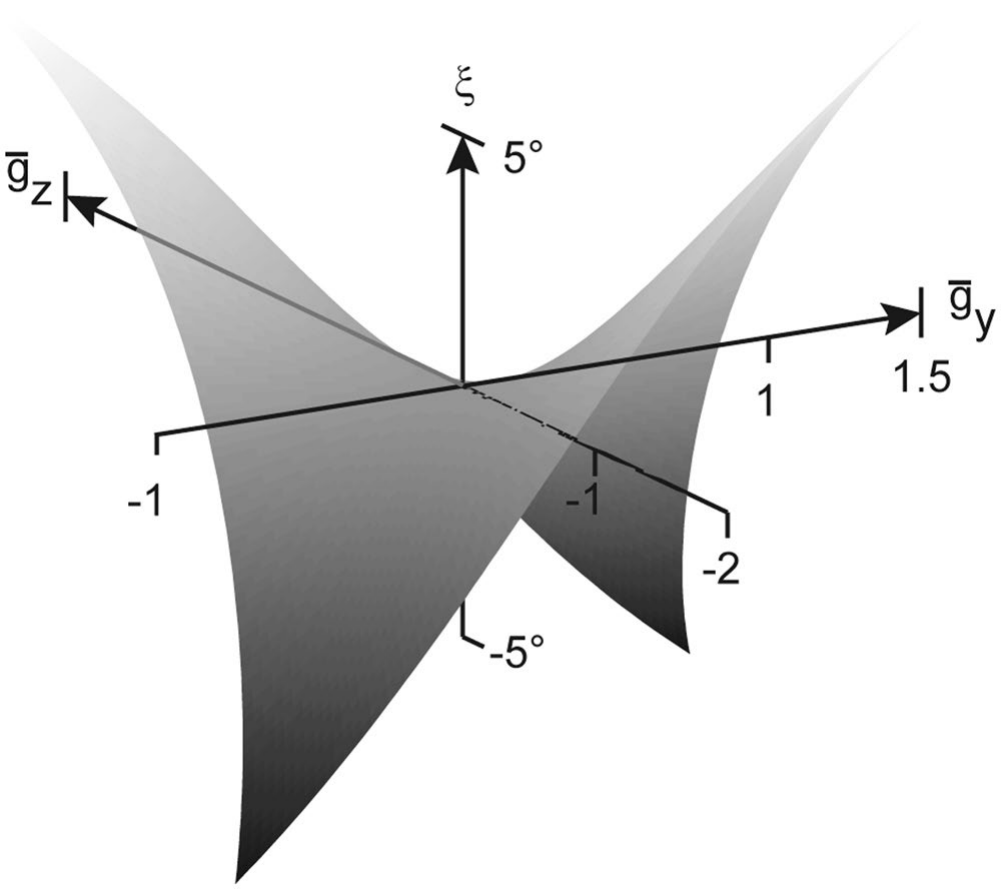
Three-dimensional plot of fusional torsion (ξ) as a function of the respective fusion positions projected onto the principal plane (denoted by ${\bar{g}}_{y}$ and ${\bar{g}}_{z}$). Note the alternating polarities of torsion in the four quadrants. Range tested ±40° × ±40°.

Since binocular single vision cannot be achieved without torsion except in primary and secondary horizontal gaze directions, we introduced the notion of torsional disparity defined as the torsional difference between Donders-Listing positions and corresponding binocular single positions of objects in near visual space (see Methods, equation 5). We shall refer to the respective ocular rotation that reduces or nullifies torsional disparity as fusional torsion. We have computed the fusional torsion across a ‘binocular’ visual field extending in horizontal and vertical direction over ±40°. We found fusional torsions of up to about ±5° at the corners of this field. The shape of the surface outlined by the torsion as a function of the projected gaze directions formed a saddle surface, that was oriented along the diagonals of the gaze field. Torsion was negative in the up-right and down-left quadrants and positive in the up-left and down-right quadrants (Fig. 5). In a large diamond-shaped flat range the torsion required for fusion was not more than about 1°. Outside this range the shape of the fusional surface becomes rather complex in terms of the two different curvatures. In paticular for asymmetric convergence, the coordination of the torsions of the eyes for fusing a near target appears to be challenging in terms of the required concerted control of different torsions of the eyes.

### Iso-torsional contours and binocular torsional disparity patterns

**Figure 6:**
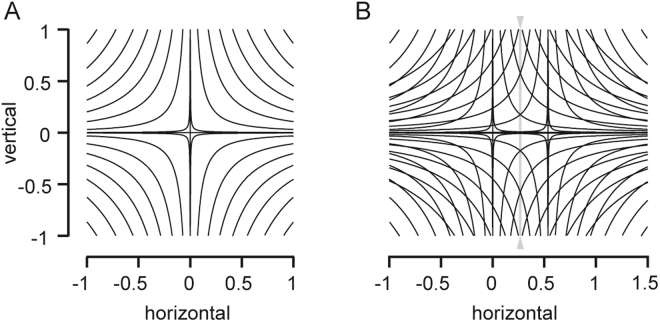
(**A**) Torsional iso-disparity contours. Each contour outlines the projected fusion positions the eye reached after having rotated through a constant angle relative to the respective Donders-Listing positions (not shown in the graph). The torsion angle of the innermost contour was 0.1° (±0.025°). All other contours were spaced evenly across the field in steps of 0.5° (relative to the center) with half-width of 0.025°. Polarity was positive in the lower left quadrant (observer looking at objects down to its left) and alternated from quadrant to quadrant. Contours did not depend on the observer’s fixation depth. (**B**) Binocular disparity map by superimposition of the right and left eye’s iso-disparity map. The location of the left eyes rotation center in right eye coordinates was $\bar{{\rm{\Delta }}}={\rm{\Delta }}/{g}_{aX}=2\,\tan \,\alpha$ evaluated at α = 15° corresponding to a convergence of 30°. In symmetric convergence the locations of binocular single vision in the visual field projected onto the H-line in the principal plane at equal distances from the rotations centers (arrowheads marking gray vertical line). Notice the symmetric intersections of iso-torsional contours along this line. In asymmetric convergence iso-torsional contours of different weights would meet at the H-line (not shown). Range tested ±40° × ±40°.

Based on the 3D torsional fusion surface we computed iso-torsion contours and fitted the data points by the same family of functions as the horizontal and vertical iso-disparity contours. We chose a torsional resolution of 0.1° at a half-width of 0.025°. The fits had mean coefficients of variation (±SD) of 0.997 (±1∙10^−3^), N = 29). The patterns of iso-torsion contours were identical for the right and left eye (Fig. 6A). For binocular single vision the required torsional fusion depended on the depth plane of fixation. Since in symmetric convergence the distances from the rotation centers to fixation points on the Helmholtz circle were identical one can readily estimate the fusional torsion of the eyes by superimposing the iso-disparity maps at the respective lateral separation conforming to the convergence angle. For example, in symmetric convergence, say of 30°, the predicted torsion of the eyes can be read of from the symmetrically overlapping iso-torsion maps as illustrated in Fig. 6B. Due to the symmetry of the patterns the iso-disparity curves that cross each other at a the H-line in the principal plane have mutually equal weights. In asymmetric vergence, the magnitudes of the right and left eye’s fusional torsion was different due to the asymmetric distances of fixation points on the Helmholtz circle to the respective rotation centers. As expected from the disparity analysis (Fig. 4A) the magnitude of fusional torsion increased with increasing vertical distance from the horizontal plane of regard. Within the central binocular field ocular torsions were opposite to each other. However, for near fixations outside the central binocular field the torsion of the ipsilateral eye changed polarity. The fusional torsion also switched polarity between the upper and lower visual hemi-field.

### Torsional fusion during far-to-near re-fixation saccades

We tested the torsional fusion model using experimental data from three Rhesus monkeys that we had been involved in earlier studies. These tests addressed two independent but related aspects of torsional fusion. First, does the model based on the angular parameters that exactly described the experimentally recorded saccades indeed yield the torsion of the eyes that would be required for perfect target fusion? For this analysis we required that the obtained fusion limit was not larger than 10^−12^. Since we found that early in the saccade the fusion model did not yield a solution because the Λ-matrix was singular (equation 3a,b), we obtained a rough estimate of the fusion position during the early saccadic time course only by a linear approximation. Secondly, we attempted to gauge the state of binocular fusion. Since a positive outcome of the first testing aspect yielded a reliable estimate of the common fusion positions of the eyes during the saccade we used the definition of disparity related to the single-eye and required $\delta {\bar{y}}_{a} < {10}^{-3}$. Since not all trials that fulfilled the first selection criterion also met the second criterion and vice versa we obtained 134 trials from 222 (36 from M1 in 2 sessions; 124 from M2 in 5 sessions; 24 trials from M2 with target up in 2 sessions; 38 trials in M3 in 2 session) which fulfilled both criteria. It should be mentioned that there were sessions with each monkey, where the overall success rate reached 80% to 100%.

**Figure 7:**
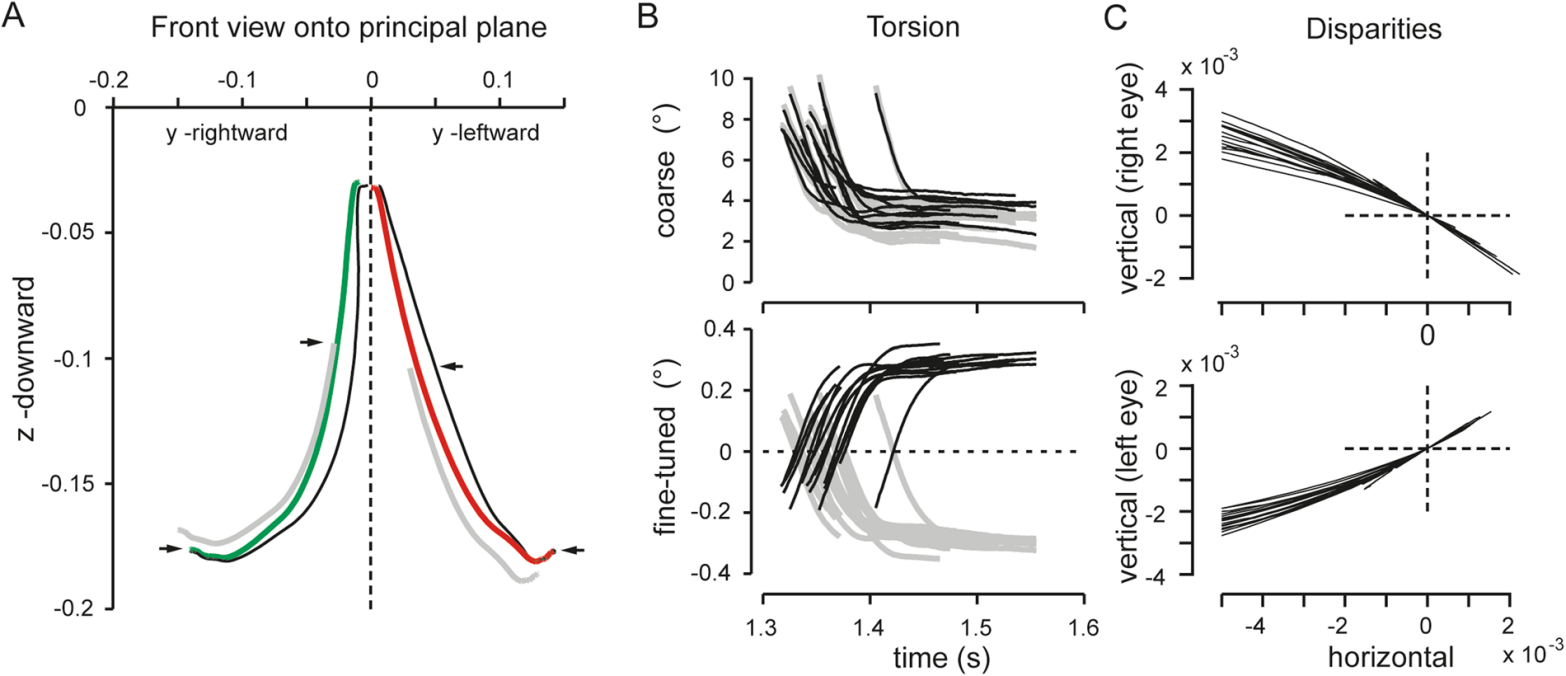
Far-to-near re-fixation saccades. (**A**) Projected trajectories of the right (red) and left (green) gaze direction during a re-fixation saccade from a far target at 80 cm straight ahead to a near target close to the median plane at ~10° down: final convergence 16.1°. Note that the left eye (green trace) moves from straight ahead (close to 0) to the right and the right eye (red trace) moves from straight ahead (close to 0) to the left. The Donders-Listing positions (gray traces) and the binocular fusion positions (black traces) were reconstructed based on the angular parameters of the saccade using equations 1a,b and 4a,b. Black traces above the arrowheads were calculated by linear approximation, final ~2/3 traces by solving the full fusion equations. Note convergence of experimental trajectories towards the predicted fusion positions at the end of the saccade (lower arrowheads). (**B**) Coarse and fine-tuned torsion of fusion solutions. Note initial conjugacy of coarse torsions (right eye: black traces, left eye: gray traces). The fine-tuned torsion was obtained by subtracting point by point the arithmetic means of the right and left eye’s coarse torsions. (**C**) Horizontal component of disparity plotted versus the vertical component during the last 50 to 100 milliseconds (right eye, top traces; left eye, bottom traces). The disparity-traces crossed the point of zero horizontal and vertical disparity towards the end of saccades, suggesting that saccadic eye positions indeed met the predicted fusion positions. All data are from the same experimental session and animal (16 trials).

In general, the torsional fusion model confirmed earlier reported findings that the eyes undergo coarse and fined-tuned torsion during far-to-near re-fixation saccades^7^, independent of whether the respective trial fulfilled the mentioned disparity criterion. Specifically, it typically yielded a solution as soon as the initially disconjugate coarse torsion approached conjugacy, which happened towards the end of the saccade. From this point onwards the fine-tuned disconjugate torsion (reported earlier also as ω-torsion^7^) started developing. In order to obtain an idea of the whole time-course, we combined the linear approximation of the early time course with the linear solution obtained towards the end of the saccade. The projection of the so computed fusion trajectory together with the experimental saccade trajectory onto the principal plane showed that the gaze lines indeed converged towards the predicted fusion positions (Fig. 7A). In a first attempt to answer the question, whether the animals indeed can fuse a point of interest in visual space such that the two gaze lines perfectly meet, we evaluated the associated single-eye horizontal and vertical disparities. Based on the time course of these disparities we found that the animals showed two different strategies of approaching the Helmholtz-point of binocular single vision. Often the disparity traces crossed one, two or even more times the zero line (crossing strategy). In other cases, the disparity simply approached or shortly contacted the zero-disparity level towards the end of the saccade (contact strategy). A graphical evaluation of the time course of the horizontal versus vertical component of disparity revealed that disparity indeed converged toward zero or crossed the zero point, sometimes also by going back and forth (Fig. 7C). The time windows in which zero (or close-to-zero) disparity was reached by the eyes varied in the range of a few tens of milliseconds or less.

To express the obtained disparities in metric terms, we combined the projective approach with the conventional Euclidean approach. We have earlier reported the estimated fusional disparities in terms of angular degrees using a mixt projective-metrical approach (see Table 3, Hess & Misslisch). Here we assessed fusion quantitatively by computing the predicted fine-tuned torsion. For this we fed the experimental gaze parameters into the fusion model to obtain the coarse fusional-torsion. Then we computed the predicted fine-tuned torsion of each eye by taking the point-by-point difference of the predicted torsion and the arithmetic mean of the predicted torsions the two eyes (see example in Fig. 7B). Finally, we compared the resulting fine-tuned torsion with the experimentally determined fine-tuned torsion, which was calibrated in angular degrees, and computed the point-by-point differences along the saccade trajectories, denoted Δ*ω*_*a*_ for the right and Δ*ω*_*b*_ for the left eye. Since these torsional trajectories were constant after about 100 ms (sessions with near target down) or 150 ms (sessions with near target up) into the saccades, finally we computed the averages of Δ*ω*_*a*_ and Δ*ω*_*a*_ over 100 ms, denoted $\overline{{\rm{\Delta }}{\omega }_{a}}$ and $\overline{{\rm{\Delta }}{\omega }_{b}}$, across all trials of sessions with M1, M2, and M3. These averages thus represented the average torsional deviations of the right and left eye from the positions that would allow the eyes to see a target point in 3D visual space single (in the mathematical sense). We found in M1: $\overline{{\rm{\Delta }}{\omega }_{a}}$ = −0.004° (±0.002°), $\overline{{\rm{\Delta }}{\omega }_{b}}$ = −0.020° (±0.004°) (N = 25 trials), in M2: $\overline{{\rm{\Delta }}{\omega }_{a}}$ = 0.04° (±0.002°), $\overline{{\rm{\Delta }}{\omega }_{b}}$ = −0.04° (±0.003°) (N = 45 trials), in M2*: $\overline{{\rm{\Delta }}{\omega }_{a}}$ = 0.05° (±0.003), $\overline{{\rm{\Delta }}{\omega }_{b}}$ = 0.03° (±0.003) (N = 27 trials) and in M3:$\overline{{\rm{\Delta }}{\omega }_{a}}$ = 0.001° (±0.002°), $\overline{{\rm{\Delta }}{\omega }_{b}}$ = −0.004° (±0.003°) (N = 30 trials). The near target was about 10° down in M1 (1 session, 1 session not included because of a constant horizontal bias), M2 (3 sessions) and M3 (2 sessions) and about 10° up in M2* (2 sessions).

## Discussion

Our main finding is that the eyes must adjust the torsional stance in order to align gaze on objects at near distances and establish binocular single vision. Although generally small in amplitude, only precision tuning of ocular torsion enables fusion of otherwise diplopic retinal images. Fusional torsion thus makes stereoscopic vision possible over a large range of gaze directions. This finding suggests an operational definition of the correspondence problem in the theory of vision: Retinal images of objects in the binocular visual field that can be fused by aligning the lines of sight and adjusting binocular torsion must be correspondent. Binocular single vision of objects located on the Helmholtz circle requires slightly different torsions dependent on vertical eye position while convergence of the eyes remains fixed. We have characterized the fusion positions for each eye across the oculomotor range by computing convergence-independent iso-torsion contours. To test the applicability of these novel concepts experimentally we provide evidence that eye positions indeed converge towards the predicted fusion positions by fine-tuned adjustments of ocular torsion at the end of far-to-near re-fixation saccades.

Iso-disparity maps of the projected retinal images were obtained by plotting the horizontal and vertical components of disparity against the projected fusion positions (Fig. 3A,B). At first sight these maps appear to be complementary in the sense that their general shape seems to be rotated relative to each other through 90°. At a closer look the contours are found to be not rotational symmetric. Indeed, the parameters of the least-squares fits of the contours were far from being reciprocal. We found that the weight of the horizontal iso-disparity contours changes more sensitively across the oculomotor range than that of the vertical iso-disparity contours.

Whereas these disparity maps per se do not depend on vergence the binocular disparities that result from combining the disparities associated to each eye (see equation 5a,b) do depend on the vergence state of the eyes. During version movements with parallel gaze directions (zero convergence), the iso-disparity maps of the right and left eye completely overlap and disparities cancel out due to opposite polarities. On the other hand, during convergence the disparities between the projected images are highly correlated. Although the notions of disparity may not be directly comparable, we like to mentioned at this point that the three-dimensional structure of a visual scene can be reconstructed in principle based on two perspective projections using horizontal and vertical disparity information alone^11,12^. On the other hand psychophysical studies have shown that the sensitivity of detecting size differences between the retinal images is at least one order of magnitude less in vertical than in horizontal direction^13^.

One of the basic notions in binocular vision is the geometric horopter defined as points in space where corresponding visual lines intersect. In far vision, correspondence of visual lines is equivalent to parallelism that is the lines intersect at optical infinity. In near visual space the criterion is equal azimuths relative to a near fixation point in the median plane. This is equivalent to stating that corresponding visual lines are those that intersect at constant vergence angle^5^. The locus of intersection points lies on the arc of a circle joining the nodal points of the eyes while passing through the fixation point. From the viewpoint of binocular visuo-motor control the criterion of constant vergence angle applies also in the vertical plane (see Fig. 1), irrespective of whether the eyes are in symmetric or asymmetric vergence^7,8^. Recently it has been proposed to extend the classical notion of retinal correspondence by first defining a mapping of corresponding points with zero disparity on the two retinas and then constructing a surface of points in visual space that project onto the two retinas at equal distance from the predefined corresponding points^6,14^. More specifically, Schreiber and colleagues showed that the classical space horopter can be extended to a surface that includes points in the binocular visual field that apparently cannot be seen single. Although considering 3D eye movements and the effect of deviations from Listing’s law^15,16^ these authors did not recognize that ocular torsions is instrumental for achieving binocular fusion^7,8^.

**Figure 8:**
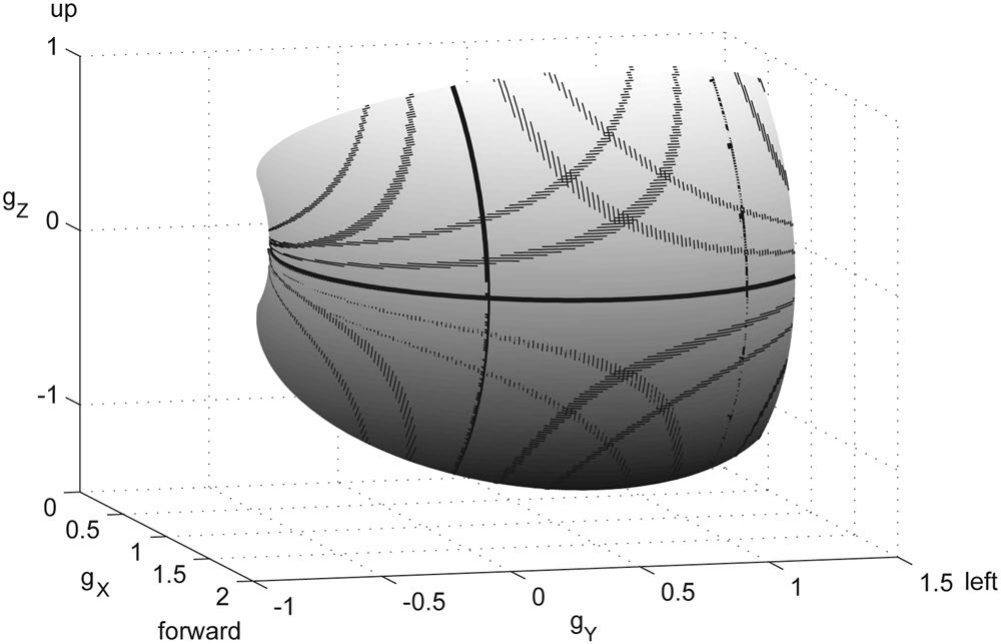
Binocular visual field in a head-fixed X-Y-Z -frame centered on the right eye (30° convergence). The rotation centers of the right and left eye were at O_a_ = (0, 0, 0) and O_b_ = (0, 1, 0). The point of symmetric convergence was at X = 1.87, Y = 0.5 and Z = 0 (1 unit = inter-ocular distance). The vertical semi-circles depict the lines of intersection of the meridian planes of each eye with the binocular visual field. The hyperbola-like curves (line width 0.2°) delineate the borders within which binocular single vison can be achieved by ocular torsions not larger than ±1° and ±2°. Range tested ±40° times ±40°.

Having calculated the torsion required for fusion of diplopic images in near vision we determined iso-torsional contour maps across the binocular visual field. These maps did not depend on the select eye since they were translational symmetric with respect to the rotation centers of the eyes. Translational symmetry implies invariance under any change of the convergence state of the eyes. At the same time, it underlines the importance of independent neuronal control of the horizontal oculomotor plant of the right and left eye^17,18^. Changes of vergence do not affect the projected fusion positions because these positions depended only on gaze eccentricity and rotations of the eye in the frontal plane. On the other hand, changes in vergence do change the depth plane of fixation by changing the lateral separation of the projected images in the principal plane. The vergence angle determines the distances to the point of intersection of the gaze lines as functions of each other eye’s azimuth, whereas the ocular rotations only adjust the directions of the line of sights. The actual location of the intersection of the gaze lines in the visual field results from the coordination of these two parameters. Based on the Donders-Listing law, the gaze lines can only intersect in the plane of the primary and secondary horizontal directions. All other locations can only be reached by adjusting the torsional stance of the eyes. Fine tuning of ocular torsion thus extends the classical one-dimensional horopter to a two-dimensional horopter surface. This surface of binocular corresponding points in the visual field is a quadratic surface, as already assumed by Helmholtz^17^ hundred and fifty years ago (Fig. 8). Its shape is basically determined by two circular arcs, one in the horizontal plane of regard that corresponds to the Vieth-Müller circle and the other in the vertical plane midway between the rotation centers. The ratio of the radii of these arcs is approximately 1 to 2. The torsional adjustments required for aligning the gaze lines on a particular location on this surface are principally within the torsional range of the eyes. For example, in the range of not more than ±1° and ±2° torsion, the locations of fusible eye positions cover the rhomboid-like surfaces outlined by the hyperbola-like curves that intersect at the meridian midway between the two rotation centers (Fig. 8). The iso-torsion contours on this surface (sketched for ±1° and ±2° torsion for each eye) cannot be obtained by intersecting it with a plane, a technique that Helmholtz used to determine his twisted cubic horopter curve^17^, because these curves have fractional exponents. Fusional torsion increases in absolute terms towards the corners of the binocular field but even in these eccentric positions amplitudes remain within the limits of the oculomotor range (Fig. 5).

At any given level of convergence, the fusional torsion allows to single out details of an object relative to its neighborhood in the ambient 3D space that otherwise would appear blurred. In the simulations presented in Fig. 8 this refers to every single point on the surface, which the observer’s attention or target-selection mechanism might focus on. Having made a saccade to the spot of interest in the peripheral visual field, the required torsional adjustments non-linearly depend on the object’s eccentricity relative to each eye’s secondary directions (see Fig. 6). We suggest that the underlying mechanisms are largely unconscious and controlled by visual feedback.

We used the here outlined algebraic approach to predict the fusion positions during far-to-near re-fixation saccades. Specifically, we asked the question whether the fusion equations can predict the torsion of each eye in flight of the saccade that would be required to align the gaze lines on the estimated location of the near target. In fact, this was possible, depending on the experimental sessions in 80% to 100% of saccades during approximately the last two thirds of the trajectories. In the early one third of the trajectory the fusion equations were singular, yielding only an approximate solution by linear approximation. The evaluation of the last third of the saccade trajectories, however, revealed three most interesting characteristics. First the solution of the torsional fusion model yielded large conjugate torsional excursions in flight of the saccade. We have earlier reported such torsion by an independent experimental approach in the same animals, which we called coarse ocular torsion^7^. At the very onset of re-fixation saccades this coarse torsion was largely disconjugate as reported. The second interesting characteristic was that the conjugate ocular torsion towards the end of the saccade turned again into dis-conjugate torsions (Fig. 7B, upper panel). The third surprising characteristic was that the fine-tuned fusional torsion of the eyes (Fig. 7B, lower panel) could be extracted by simple arithmetic manipulations from the predicted torsions yielding perfect fusion (in mathematical terms). Since we found that the earlier experimentally-derived fine-tuned torsion (called ω-torsion, expressed in each eye’s coordinate system, Hess and Misslisch^7^) was statistically indistinguishable from the predicted fine-tuned fusional torsion (here expressed in right-eye centered coordinates), we conclude that it is this late-onset dis-conjugate torsion that ultimately leads to fusion of the diplopic target images. The relatively slow and late onset of the convergence of the gaze lines towards meeting at a single spot in 3D visual space suggests that convergence is driven by visual feedback. Similar conclusions have been drawn from studies of cyclovergence during steady fixations in humans^19^.

An intriguing aspect arising from these analyses and our earlier experiments^7,8^ is what function the conjugate coarse torsion during far-to-near re-fixation saccades might have. From geometric studies of the laws correlating two or more views of a visual scene, it is known that corresponding image points lie in general on epi-polar lines, which are related to each other by quadratic constraints. If these images are generated by parallel projection as is the case of conjugate eye movements, the epi-polar lines and planes are all parallel because the quadratic constraint relaxes to a linear constraint^20,21^. In this situation it is possible to extract from images that are cyclo-rotated relative to each other not only affine but also metric information including depth information about the visual scene^22^.

## Electronic supplementary material

Supplementary Material
