# Publisher Correction: On the role of ocular torsion in binocular visual matching

**DOI:** 10.1038/s41598-019-49308-5

**Published:** 2019-09-10

**Authors:** Bernhard J. M. Hess

**Affiliations:** Department of Neurology, University Hospital Zurich, Zurich, CH-8091 Switzerland

Correction to: *Scientific Reports* 10.1038/s41598-018-28513-8, published online 13 July 2018

This Article contains typographical errors in the Discussion section where,

“This surface of binocular corresponding points in the visual field is a quadratic surface, as already assumed by Helmholtz^17^ hundred and fifty years ago (Fig. 8).”

should read:

“This surface of binocular corresponding points in the visual field is a quadratic surface, as already assumed by Helmholtz^23^ hundred and fifty years ago (Fig. 8).”

Additionally,

“The iso-torsion contours on this surface (sketched for ±1° and ±2° torsion for each eye) cannot be obtained by intersecting it with a plane, a technique that Helmholtz used to determine his twisted cubic horopter curve^17^, because these curves have fractional exponents.”

should read:

“The iso-torsion contours on this surface (sketched for ±1° and ±2° torsion for each eye) cannot be obtained by intersecting it with a plane, a technique that Helmholtz used to determine his twisted cubic horopter curve^23^, because these curves have fractional exponents.”

In addition, this Article contains an error in Equation 1b.

$${\hat{g}}_{b}={R}_{F}{\hat{d}}_{b}{R}_{F}^{-1}={Q}_{b}{\hat{e}}_{1}+\{{S}_{b}\,\cos \,{\xi }_{b}+{R}_{b}\,\sin \,{\xi }_{b}\}{\hat{e}}_{2}-\{{R}_{b}\,\cos \,{\xi }_{a}-{S}_{b}\,\sin \,{\xi }_{b}\}{\hat{e}}_{3}$$

should read:

$${\hat{g}}_{b}={R}_{F}{\hat{d}}_{b}{R}_{F}^{-1}={Q}_{b}{\hat{e}}_{1}+\{{S}_{b}\,\cos \,{\xi }_{b}+{R}_{b}\,\sin \,{\xi }_{b}\}{\hat{e}}_{2}-\{{R}_{b}\,\cos \,{\xi }_{b}-{S}_{b}\,\sin \,{\xi }_{b}\}{\hat{e}}_{3}$$

