# Supplementary material for: On the role of ocular torsion in binocular visual matching
Source: Sci Rep. 2018 Jul 13;8:10666. doi: 10.1038/s41598-018-28513-8 (PMC6045635; doi:10.1038/s41598-018-28513-8)
Supplement: Supplementary file 1 — Supplementary Material [file 41598_2018_28513_MOESM1_ESM.pdf]

On the role of ocular torsion in binocular visual matching

Bernhard J. M. Hess
Department of Neurology, University Hospital Zurich, Zurich CH-809, Switzerland

Supplementary information

To evaluate rotations, we used the Clifford algebra of the 3D-Euclidean space, which is generated by three numbers, labelled $\hat{\gamma}_1, \hat{\gamma}_2, \hat{\gamma}_3$ and a unity denoted *I*. These numbers are defined by the properties $\left(\hat{\gamma}_i\right)^2 = I$ (identity) and $\hat{\gamma}_j\hat{\gamma}_k + \hat{\gamma}_k\hat{\gamma}_j = 2\delta_{jk}I$ with $\delta_{jk} = 1$ for j =k and $\delta_{jk} = 0$ if j ≠k, whereby the indices i, j, k run from 1to 3 (Snygg 1997). Euclidian vectors can be represented in this algebra by identifying the Cartesian basis vectors $\hat{e}_i$ by the Clifford numbers $\hat{\gamma}_i$ (i=1, 2, 3), also called basis 1-vectors. A rotation of a 1-vector x is obtained by the conjugation $R_A x R_A^{-1} = x'$ with the operator $R_A = I\cos(\chi/2) - \sin(\chi/2)\hat{\gamma}_{\alpha\beta}$ where χ is the angle of rotation in the plane $\hat{A} = \hat{\gamma}_{\alpha\beta}$ spanned by the 1-vectors $\hat{\gamma}_\alpha$ and $\hat{\gamma}_\beta$ ($|\hat{A}|=1$). The inverse of R_A is $R_A^{-1}\left(\chi\right) = R_A\left(-\chi\right)$.

General eye positions

A general eye position can be described by the following compounded rotation $R = R_F(\xi)R_v(\eta)R_H(\vartheta) = R_F(\xi)R_{DL}(\eta,\vartheta)$, where R_F describes a rotation in the frontal plane through the angle ξ, and $R_{DL}(\eta,\vartheta) = R_v(\eta)R_H(\vartheta)$ is a torsion-free or Donders-Listing rotation through the angles ϑ and η in the horizontal and vertical direction-plane. The gaze direction associated with such rotation is $\hat{g} = R_F R_{DL}\hat{g}_0 R_{DL}^{-1}R_F^{-1}$ where $\hat{g}_0$ denotes the reference direction. To evaluate this expression, we identified the Cartesian vectors $\hat{e}_i$ (i =1, 2, 3) with the 1-vectors $\hat{\gamma}_i$ (i =1, 2, 3) and the frontal, vertical and horizontal plane with the 2-vectors $\hat{\gamma}_{23} := \hat{\gamma}_2\hat{\gamma}_3$, $\hat{\gamma}_{31} := \hat{\gamma}_3\hat{\gamma}_1$ and $\hat{\gamma}_{12} := \hat{\gamma}_1\hat{\gamma}_2$, respectively. Using $R^n = \left(I\cos\alpha/2 + \sin\alpha/2\,\hat{\gamma}_{\alpha\beta}\right)^n = I\cos\left(n\alpha/2\right) + \sin\left(n\alpha/2\right)\hat{\gamma}_{\alpha\beta}$ we note first that

$$R_F R_{DL}\hat{\gamma}_1 R_{DL}^{-1}R_F^{-1} = \hat{\gamma}_1 R_F R_{DL}^{-2}R_F^{-1} \qquad (1)$$

based on the identity $R_F R_{DL}\hat{\gamma}_1 = \hat{\gamma}_1 R_F R_{DL}^{-1}$. To see this, we evaluated the compounded rotation $R_{DL} = R_v R_H$ by taking care of rotating the vertical direction-plane due to the preceding horizontal rotation. With $\hat{\gamma}_{vDL} = R_H\hat{\gamma}_{31} = -\sin\vartheta/2\,\hat{\gamma}_{23} + \cos\vartheta/2\,\hat{\gamma}_{31}$ we obtain

$$\begin{aligned} R_{DL} = R_v R_H &= \left(I\cos\eta/2 - \sin\eta/2\,\hat{\gamma}_{vDL}\right)\left(I\cos\vartheta/2 - \sin\vartheta/2\,\hat{\gamma}_{12}\right) \\ &= I\cos\eta/2\cos\vartheta/2 - \sin\eta/2\,\hat{\gamma}_{31} - \cos\eta/2\sin\vartheta/2\,\hat{\gamma}_{12}. \end{aligned} \qquad (2)$$

The result (1) now follows from the observation that $R_{DL}\hat{\gamma}_1 = \hat{\gamma}_1 R_{DL}^{-1}$ and $R_F\hat{\gamma}_1 = \hat{\gamma}_1 R_F$.

Evaluation of $R_{DL}^{-2} = R_H^{-2}R_v^{-2}$ yields $R_{DL}^{-2} = IQ + R\hat{\gamma}_{31} + S\hat{\gamma}_{12}$ with the coefficients

$Q=\cos\vartheta\cos^2\eta/2-\sin^2\eta/2$, $R=\cos\vartheta/2\sin\eta$ and $S=\sin\vartheta\cos^2\eta/2$. Using the general rule $R_A^n(\rho)=R_A(n\rho)$, $R_F\hat{\gamma}_2=\hat{\gamma}_2R_F^{-1}$ and $R_F\hat{\gamma}_3=\hat{\gamma}_3R_F^{-1}$, we obtained for a general eye position

$$\begin{aligned}\hat{\gamma}_1' &= \hat{\gamma}_1 R_F R_{DL}^{-2} R_F^{-1} = R_F\left(Q\hat{\gamma}_1+S\hat{\gamma}_2-R\hat{\gamma}_3\right)R_F^{-1}\\ &= Q\hat{\gamma}_1+\left(S\cos\xi+R\sin\xi\right)\hat{\gamma}_2+\left(S\sin\xi-R\cos\xi\right)\hat{\gamma}_3.\end{aligned} \tag{3}$$

Ocular torsion enables single binocular vision in general eye positions

We now determined the set of single binocular fixation positions based on equation (3) that describes the general gaze direction of the right and left eye. We have earlier found that the distances from the rotation centers to a common fixation point are $a=\cos\beta/\sin(\alpha-\beta)$ for the right and $b=\cos\alpha/\sin(\alpha-\beta)$ for the left eye. In the horizontal plane of regard, the angles α and β coincide with the horizontal rotation angles ϑ_a and ϑ_b (see Fig. 1). To distinguish the formulas for the right and left eye, we use subscripts 'a' and 'b'. For the gaze-line coordinates, we shall use superscripts 'a' and 'b' in parentheses. We obtained the following set of equations of the gaze vectors $\mathbf{g}_a$ and $\mathbf{g}_b$, expressed in Cartesian coordinates.

$$g_1^{(a)}=aQ_a,\ g_2^{(a)}=a\left(R_a\sin\xi_a+S_a\cos\xi_a\right),\ g_3^{(a)}=-a\left(R_a\cos\xi_a-S_a\sin\xi_a\right) \tag{4a}$$

$$g_1^{(b)}=bQ_b,\ g_2^{(b)}=b\left(R_b\sin\xi_b+S_b\cos\xi_b\right),\ g_3^{(b)}=-b\left(R_b\cos\xi_b-S_b\sin\xi_b\right) \tag{4b}$$

A first geometric condition for target fusion is that $g_1^{(a)}=aQ_a=bQ_b=g_1^{(b)}$. After a few algebraic manipulations, this identity leads to the following constraint on the elevation angles η_a, η_b of the right and left eye.

$$\eta_b=2\sin^{-1}\left\{\sqrt{\frac{\cos\beta(\cos\alpha+1)}{\cos\alpha(\cos\beta+1)}}\sin\eta_a/2\right\}=2\sin^{-1}\left\{\frac{\cos\alpha/2}{\cos\beta/2}\sqrt{\frac{\cos\beta}{\cos\alpha}}\sin\eta_a/2\right\} \tag{5}$$

This relation implies that the ratio between the elevations is unity in conjugate eye movements as well as in symmetric convergence. Notice that such a segregation of the elevation as a function of the eyes' azimuths is not possible in Helmholtz coordinates.

A second constraint is that the torsional rotation angles of the right and left eye must fulfill the following equations.

$$a\begin{pmatrix}S_a\cos\xi_a+R_a\sin\xi_a\\ -R_a\cos\xi_a+S_a\sin\xi_a\end{pmatrix}=b\begin{pmatrix}S_b\cos\xi_b+R_b\sin\xi_b\\ -R_b\cos\xi_b+S_b\sin\xi_b\end{pmatrix}+\begin{pmatrix}1\\0\end{pmatrix}. \tag{6}$$

On one hand, we derived from these linear equations

$$\begin{pmatrix}\sin\xi_a\\ \cos\xi_a\end{pmatrix} = p_a\begin{pmatrix}\cos\sigma_{ba} & \sin\sigma_{ba}\\ -\sin\sigma_{ba} & \cos\sigma_{ba}\end{pmatrix}\begin{pmatrix}\sin\xi_b\\ \cos\xi_b\end{pmatrix} + q_a\begin{pmatrix}R_a\\ S_a\end{pmatrix}, \qquad (7a)$$

with $D = \left(R_a^2 + S_a^2\right)\left(R_b^2 + S_b^2\right)$, $\cos\sigma_{ba} = \left(R_b R_a + S_b S_a\right)\big/\sqrt{D}$, $\sin\sigma_{ba} = \left(S_b R_a - R_b S_a\right)\big/\sqrt{D}$,
$p_a = (b/a)\sqrt{D}\big/\left(R_a^2 + S_a^2\right)$ and $q_a = (1/a)\big/\left(R_a^2 + S_a^2\right)$.

On the other hand, from the same two linear equations we derived

$$\begin{pmatrix}\sin\xi_b\\ \cos\xi_b\end{pmatrix} = p_b\begin{pmatrix}\cos\sigma_{ab} & -\sin\sigma_{ab}\\ \sin\sigma_{ab} & \cos\sigma_{ab}\end{pmatrix}\begin{pmatrix}\sin\xi_a\\ \cos\xi_a\end{pmatrix} - q_b\begin{pmatrix}R_b\\ S_b\end{pmatrix}, \qquad (7b)$$

with $\sigma_{ab} = \sigma_{ba}$, $p_b = (a/b)\sqrt{D}\big/\left(R_b^2 + S_b^2\right)$ and $q_b = (1/b)\big/\left(R_b^2 + S_b^2\right)$.

Finally, we obtained from equation (7a)

$$\sin\left(\xi_b + \sigma_{ba} + \sigma_a\right) = \sin\left(\xi_b + \sigma_b\right) = \left\{1 - p_a{}^2 - q_a{}^2\left(R_a{}^2 + S_a{}^2\right)\right\}\big/ 2 p_a q_a\sqrt{R_a{}^2 + S_a{}^2} , \qquad (8a)$$

with $\sin\sigma_a = S_a\big/\sqrt{R_a^2 + S_a^2}$, $\cos\sigma_a = R_a\big/\sqrt{R_a^2 + S_a^2}$.

And from equation (7b)

$$\sin\left(\xi_a - \sigma_{ab} + \sigma_b\right) = \sin\left(\xi_a + \sigma_a\right) = \left\{1 - p_b{}^2 - q_b{}^2\left(R_b{}^2 + S_b{}^2\right)\right\}\big/\left(2 q_b p_b\sqrt{R_b{}^2 + S_b{}^2}\right) \qquad (8b)$$

with $\sin\sigma_b = S_b\big/\sqrt{R_b^2 + S_b^2}$ and $\cos\sigma_b = R_b\big/\sqrt{R_b^2 + S_b^2}$.

Several conclusions can be derived from the equations 5 to 8. Firstly, the torsion of each eye during fusion depends on the azimuth and elevation of the other eye. Secondly, the torsional angles ξ_a and ξ_b of equations 8a and 8b solve the linear equations 6 that express the geometric constraint for aligning the gaze lines on a single object in secondary vertical and tertiary eye positions. Thirdly, in conjugate gaze (α =β) the elevation of the eyes must be equal ($\eta_a = \eta_b$) that is the eyes must move in conjugation in parallel planes. Moreover, it follows also that the torsion of the eyes must be equal and remain invariant, independently of the conjugate motion.

Gaze lines are co-planar during fusion

Equations (4), (5) and (7) describe the conditions for the intersection of the gaze lines at a single point when the eyes are in general positions. We now check that these equations imply co-planarity of the gaze lines, which is a necessary condition for intersection of the gaze lines during fusion. Firstly, we notice that the Donders-Listing positions are in general not coplanar except for version movements (α =β) or symmetric convergence of the eyes (α =-β) where the ratio $\sin\left(\eta_a/2\right)\big/\sin\left(\eta_b/2\right) = 1$ (equation 5). In the general case, we must show that the scalar triple product $\hat{g}_a \times \hat{g}_b \bullet \hat{e}_2 = g_3^{(a)} g_1^{(b)} - g_1^{(a)} g_3^{(b)}$ is zero if the equations (4), (5) and (8) are fulfilled. Using equations (4) the condition is

$$Q_b \begin{pmatrix} S_a \\ -R_a \end{pmatrix} \cdot \begin{pmatrix} \sin\xi_a \\ \cos\xi_a \end{pmatrix} = Q_a \begin{pmatrix} S_b \\ -R_b \end{pmatrix} \cdot \begin{pmatrix} \sin\xi_b \\ \cos\xi_b \end{pmatrix}.$$

Introducing equation (7a) on the left side and using the definition of Λ-matrix we obtain

$$\begin{pmatrix} S_a \\ -R_a \end{pmatrix} \cdot \Lambda \begin{pmatrix} \sin\xi_b \\ \cos\xi_b \end{pmatrix} = \Lambda^{-1} \begin{pmatrix} S_a \\ -R_a \end{pmatrix} \cdot \begin{pmatrix} \sin\xi_b \\ \cos\xi_b \end{pmatrix} = \begin{pmatrix} S_b \\ -R_b \end{pmatrix} \cdot \begin{pmatrix} \sin\xi_b \\ \cos\xi_b \end{pmatrix}$$

. Hereby we used equation (5) and the property that the inverse of unitary operator Λ is its adjoint: $\boldsymbol{x} \cdot \Lambda \boldsymbol{y} = \Lambda^{-1} \boldsymbol{x} \cdot \boldsymbol{y}$. One easily verifies that $\Lambda^{-1}(S_a, -R_a)^T = (S_b, -R_b)^T$ by using the relations defining the Λ-matrix $\cos\sigma_{ba} := (R_b R_a + S_b S_a)/\sqrt{D}$ and $\sin\sigma_{ba} := (S_b R_a - R_b S_a)/\sqrt{D}$.

Supplementary Table

Table S1: Zero disparity constraints on Donders-Listing eye movements, i.e. on eye movements that leave torsional stance of the eyes invariant. Abbreviations: α, β: azimuths of right, left eye; η_a, η_b: elevations of right, left eye (measured in the respective direction planes);

	Horizontal disparity $\delta\bar{y} = 0$	Vertical disparity $\delta\bar{z} = 0$
General constraints	$\cos\beta \sin\alpha \cos^2\eta_a/2 - \cos\alpha \sin\beta \cos^2\eta_b/2$ $= \sin(\alpha - \beta)$	$\cos\beta \cos\alpha/2 \sin\eta_a$ $= \cos\alpha \cos\beta/2 \sin\eta_b$
A. Conjugate Donders-Listing eye movements ($\alpha = \beta$ & $\alpha \neq 0$)		
Constraints	$\cos\beta \sin\alpha \left(\cos^2\eta_a/2 - \cos^2\eta_b/2\right) = 0$ ⇒equal elevations ($\eta_a = \eta_b$)	$\sin\eta_a = \sin\eta_b$ ⇒equal elevations ($\eta_a = \eta_b$)
B. Dis-conjugate Donders-Listing eye movements		
symmetric α =-β	$\left(\cos^2\eta_a/2 + \cos^2\eta_b/2\right) = 2$ ⇒ zero elevations ($\eta_a = \eta_b = 0$)	$\sin\eta_a = \sin\eta_b$ ⇒equal elevations ($\eta_a = \eta_b$)
asymmetric |α|≠|β|	general constraint ⇒zero elevations ($\eta_a = \eta_b = 0$)	general constraint ⇒zero elevations ($\eta_a = \eta_b = 0$)

Reference

Snygg J. *Clifford Algebra – A Computational Tool for Physicists*. New York: Oxford UP, 1997.
